# Honoring Black Hopes: How to respond when the family is hoping for a miracle

**DOI:** 10.12688/f1000research.109811.1

**Published:** 2022-03-02

**Authors:** John Stonestreet

**Affiliations:** 1 Hopecare, 740 4th St. N, Saint Petersburg, Florida, 33701, USA

**Keywords:** End-of-life disparities, prognosis communication, goals of care, belief in miracles, spiritual intervention

## Abstract

**Background:** Racial and ethnic disparities in end-of-life healthcare can be reduced by showing physicians how to best respond to a documented underlying cause: African American families’ hopes for a miracle via divine intervention influence their end-of-life medical decisions, like, for example, making them not want to withdraw ventilatory support in cases of poor neurologic prognosis because they are still hoping for God to intervene.

**Methods:** Autoethnographic research probing the author’s Spiritual Care experience in this context yields a nuanced, 90-second point-of-care spiritual intervention physicians can use to address the religious aspect of African American families who base end-of-life medical decisions on their hopes for a miracle via divine intervention. Autoethnographic analysis is framed by physician-author, Dr. Jessica Zitter’s documented journey of grappling with this context. The evolution of Dr. Zitter’s responses to miracle-hoping African American families provides a framework for applying autoethnographic analysis to a critical appropriation of the Johns Hopkins “AMEN” communication protocol for families hoping for a miracle.

**Results: **The common instinct of white physicians to remain neutral, holding miracle-hoping African American families at arm’s length, rather than supportively engaging their hopes, is shown to be an intellectual ruse for emotional avoidance. A novel, counterintuitive spiritual intervention for the religious aspect of miracle-hoping African American families is integrated into an existing physician communication protocol for responding to families hoping for a miracle with recommendations for utilization of existing communication technology when necessary.

**Conclusion: **Properly addressing the religious dimension of African American families hoping for a miracle may help physicians to increase their therapeutic connection with families, decrease their own stress/burnout levels, and eliminate racial and ethnic disparities in end-of-life healthcare.

## Introduction

While most health disparities for African Americans result in receiving less medical care, this trend changes at the end of life, ^1^ when African Americans often receive more aggressive interventions than white patients. ^1^^–^^3^ But at this juncture, more tends not to be better because aggressive measures at the end of life lead to more distress for both patients and families who then have more to gain from bio-psycho-social-spiritual comfort. ^1^^,^^4^^,^^5^ These disparities in extra burdensome, death-prolonging care have been studied as legacies of slavery and racial discrimination. ^6^ End-of-life conversations with physicians have been shown to decrease harmfully aggressive measures for white patients but less so for African Americans. ^7^^,^^8^ That may be an issue of trust. Researchers have identified patient/family trust in physicians as a critical issue for end-of-life disparities. ^9^ In addition to well-known histories of medical racism, one trait that has been shown to decrease patient/family trust in physician prognosis is belief in miraculous healing by divine intervention. ^10^^,^^11^ Indeed, it seems that racial disparities may sometimes boil down to religious issues. ^1^^,^^12^ In statistical analysis, religious beliefs (including, the most salient belief that “God can perform a miracle and cure me”) have been found to account for racial disparities between African American and white patients in end-of-life healthcare. ^13^ And belief in miracles is independently associated with a more complicated death. ^14^ This means that addressing racial and ethnic disparities in end-of-life healthcare requires spiritual interventions where physicians address the underlying religious issues; however, this is not an easy task.

Physician-family conflicts over beliefs in miracles by divine intervention are too often characterized by ineffective communication, “resulting in the patient/family feeling disrespected and a frustrated clinical team”. ^15^ Often, this is because patient/family miracle hopes are “not tolerated” by the medical team, ^16^ perhaps because they statistically alter families’ prognosis interpretation in contradiction to scientific evidence. ^17^^,^^18^ It is found that religious belief in miracles underlying racial and ethnic disparities in end-of-life healthcare is not adequately addressed by physicians. ^16^ As a result, African Americans are more likely to overestimate their life expectancy than white patients. ^19^ Experts suggest that physicians assess patients and families for religious belief in miracles and tailor prognosis communication accordingly. ^17^^,^^18^ But this tailored communication can devolve into bitter arguments because some physicians take an adversarial approach with miracle-hoping families, dismissing them as “irrational,” saying things like, “Well God also instructed us in medicine …. We’re not working against God”. ^16^ Less direct forms of disagreement over this issue tend to come in the form of patient and/or family avoidance by physicians, which has been known to cause complicated grief in the African American family members (one wife ended up almost losing her will to live), ^20^ and burnout among physicians, another outcome that is detrimental to the extreme. ^21^ But even when physicians approach such conversations with African American families openly and earnestly, these exchanges still tend to be strained at best. ^22^

This may mean that it is not enough for physicians to be open to families’ religious beliefs; perhaps physicians must also find ways to be affirming in these conversations with African American patients and families, to bridge relational gaps and build the trust required for truly effective physician-patient/family communication. But what would that even look like, and how could it be trained? To address this common physician-patient/family communication impasse underlying racial disparities in end-of-life healthcare, it is helpful to understand: 1) what physicians and African American families are each bringing to the table when they come into conflict over the families’ belief in miracles, and 2) whether and how well these issues are addressed by existing physician communication protocols for this context.

The award-winning short documentary film, *Extremis,* ^22^ paints a vivid picture of what may be one of the better case scenarios for status quo physician-family conflicts over African American families’ beliefs in miracles via divine intervention and the impact these beliefs have on their end-of-life medical decisions. The film follows Dr. Jessica Zitter and her team through the intensive care unit (ICU) at Highland Hospital in Oakland, California, as they seek to help families make medical decisions for their loved ones facing death. While Dr. Zitter started her medical career focused exclusively on rescuing patients from the brink of death, she is now equally passionate about rescuing the dying from an unnecessarily drawn out, medicalized death that they may not have wanted. ^23^ In the ICU environment, this often involves securing the family’s agreement to turn off the ventilator that is breathing for the patient and allow nature to take its course (a procedure known as compassionate extubation) which is a difficult decision for many families to make. As the documentary conveys, it is especially difficult when the family hopes for a miracle by divine intervention. This hope in God for another sort of miracle when medicine has reached the end of its own miracle promises is perhaps one of the most challenging issues for physicians to negotiate with the families of dying patients. What is the physician to say?

Speaking to the brothers of a dying African American patient named Selena, Dr. Zitter invites them into her world by sharing how emotionally painful the situation is for her as a physician:

Every day people with very poor neurologic prognoses are attached permanently to machines, and, unfortunately, it’s very hard, emotionally, for us physicians when we feel that we’re taking a body and we’re just keeping it alive when it’s not really the person. ^22^

After hearing how his sister’s subsistence on a breathing machine is affecting Dr. Zitter, one of Selena’s brothers responds in turn by inviting Dr. Zitter into his world: “But God has proven to me, miracles are miracles, and we’re askin’ him for one”. ^22^ Selena’s brother was not the only member of this African American family hoping for a miracle. Dr. Zitter has a similar conversation with Selena’s daughter who, like her uncle, is hoping for a miracle: “To me the whole situation is miraculous,” she said, “ … so I’m always looking for another miracle.” Meeting Selena’s daughter on her own terms, Dr. Zitter assures her that the whole medical team is also hoping for a miracle. But Selena’s daughter doesn’t seem convinced, and this naked assertion draws no response. Despite her efforts, Dr. Zitter’s conversation with Selena’s brothers and daughter still seems strained. It is almost as if Dr. Zitter is pulling on one end of a rope and Selena’s brothers and daughter are holding fast to the opposite end. But this communication impasse is not for lack of effort by either party. In both of these conversations, Dr. Zitter seems to be doing everything she can to connect authentically with this family in sincerity. She is respectfully vulnerable and transparent about her own moral distress over artificially extending death, and she even meets them on their own terms by affirming their hope for a miracle. But somehow, the two parties still seem engaged in a tension-filled tug-of-war. When healthcare decision-making turns into a tug-of-war between physician and family, unfortunately neither side wins.

In the case of Dr. Zitter and Selina’s family, the documentary ends by stating that “Selena was surgically attached to the breathing machine” where she “regained periods of consciousness before passing away six months later”. ^22^ What may be more regrettable than the medical outcome (which is still subject to interpretation) is the lack of a therapeutic connection between doctor and family who seem like trains passing in the night. Is there anything more Dr. Zitter could have said or done to change the tenor of the conversation? When medical science intersects with non-medical issues like patient/family religious beliefs, how are physicians to navigate these murky waters?

## Methods

Because physicians do not specialize in this area, and innovative suggestions are scarce, this article utilizes the qualitative research method of autoethnography ^24^ to grant physicians access into the sociological domain in which patients and families are operating when their religious beliefs in miracles determine their medical decisions for intensive, life-prolonging care. As a hospital Spiritual Care Provider, I have never been in a physician’s position to make a medical case for a family to consider removing a ventilator and allowing a more natural death. However, many African American families have asked me to pray for miraculous healing (against all odds), and I believe that my journey of struggling to respond appropriately to the religious aspect of miracle-hoping families may shed some light on physician communication in this challenging context. I therefore offer a critical analysis of my Spiritual Care experience with miracle-hoping African American families, interpreted through the lens of the leading physician communication protocol for this context, as autoethnographic research data for the many physicians who find themselves at a loss for how to respond to this religious aspect of healthcare decisions underlying racial and ethnic disparities in end-of-life healthcare.

## Results

Although a number of years have passed, I can still remember the first time I was asked to pray for a miracle in my Spiritual Care internship at Duke University Hospital. The patient’s husband said:

The doctor says she’s going to die, but we know that a higher power determines that, and we believe in miracles. So, we want you to pray for a miracle.

That simple request was deeply troubling to me. My first thought was:

If a Duke doctor says she’s going to die, then I don’t want to pray for her in a way that causes or increases the family’s denial and makes it harder for them to come to grips with reality and say their goodbyes before it’s too late. But I also don’t feel comfortable just blowing off their earnest request. And I do share their hope that she defies the odds. But I pretty much feel “damned if I do” (pray for their miracle) “and damned if I don’t.”

Suffice to say, I felt tremendous anxiety and ambivalence about how to respond.

The following day, I remember doing a joint visit in the ICU with a divinity school student who didn’t seem to share my ambivalence. During our visit, parents of a young man with late-stage terminal cancer asked her to pray for a miracle, and she didn't seem to have any of that hesitation or anxiety/ambivalence that I felt; she just launched right into a passionate, wholehearted prayer for their miracle. A part of me was jealous of her ability to respond so readily and sincerely. If the gold standard of healthcare communication is person-centered care, wasn’t that precisely what she was providing in that moment? There the parents were hoping in God for the impossible, and there she was, right with them, praying for that very miracle. A part of me thought, “Wow! That’s beautiful. Nothing is holding her back from being down in the trenches with them right where they are.” Another part of me felt just the opposite; that part of me felt deeply cynical. I can still recall some of the words of her prayer. She said:

And God, we just ask you to do what you do best, and that is provide health and healing! And we thank you in advance for what you will do and for what you are doing at this very moment!

I remember feeling a deep cynicism at that moment, thinking:

Well, if that’s what God does best, then he's doing a really bad job here at Duke Hospital! He's either lazy, or he's unmotivated, or he just doesn’t care, because people are praying for miracles and dying in here left and right.

My divinity school colleague’s presumptive prayer made me not only cynical but morally distressed. I wanted to ask:

Wait a second, what is it that we are promising here, and how can we just make a declaration like that and waltz out of this hospital room as if everything's going to be fine? As if we know our prayer had been meaningful in the way that it was intended. On the contrary, we know that in most cases in these situations, God does not do what we just said he does best.

I continued to be bothered by that experience, as I struggled to reconcile my admiration with my cynicism and moral distress. It seemed to me that there was something so right and something so wrong about that prayer.

My divinity school colleague was not alone in approaching prayer for unlikely outcomes with a disconcerting assumption of their likelihood. Although atypical among 21st century hospital chaplains, a chaplain-author working in the 1980s articulated a similar approach. ^25^ Chaplain Furniss counseled patients to pray once for their own physical healing, and then revert to “giving thanks in faith for the [physical] healing that is [already] happening” since “Jesus says, ‘Ask and you shall receive’”. ^25^ Furniss did qualify his recommendations for healing prayer, conceding that, “Where advanced age is present or the will to live is gone, I do not think it is necessary for us to engage in healing prayer.”

But what is the threshold for “advanced age?” And what does this mean for cases where advanced age is not present or the will to live is not gone? Are we to assume that earnest prayer will usually overcome terminal illness for the young and/or hopeful? As a Spiritual Care Provider who has seen the full spectrum of death, from working in adult hospice, to working at Johns Hopkins All Children’s Hospital, it is an understatement to say that I would not feel comfortable counseling children and families facing a grim prognosis that they should simply pray for physical healing and then assume a successful outcome. Jesus himself prayed “let this ‘cup’ pass from me” but he did not conclude the prayer with an assumption that the “cup” was already passing, and indeed…the “cup” did not pass.

Returning to my early recollection of peer responses to families hoping for a miracle, I remember another occasion that made a lasting impression on me. One of my chaplain-in-training colleagues at Duke shared in group/class that he was asked to pray for a miracle earlier that day in a case where a wife and daughter did not want to let go of their dying 72-year-old husband/father with multiple comorbidities. My colleague reported that he had to tell the family that praying for miracles wasn’t something he could do because it didn’t fit with his beliefs. Instead, he offered to journey with them in their suffering and help them to prepare for a good death.

Once again, I experienced a colleague’s response to hope for miraculous healing with ambivalence. But this time, a part of me really admired my colleague for facing the most likely reality of their situation, being honest about it, and being willing and able to walk down that path with them. At the same time, another part of me was deeply saddened. If our goal in healthcare is person-centered care, my colleague had provided the opposite. A family had asked him from the depths of their hearts for what they most yearned for, but he dismissed their request as something too incompatible with his beliefs. It seemed to me then—and seems to me still—that the family’s request should not be so quickly dismissed. How could he just opt out of meeting people where they were and joining together with them in bringing their hopes to God? His inability to do that made me wonder if they felt comfortable enough with him to consider his counter-offer. Even if they had at least appeared to accept it, could their acceptance have been genuine?

My chaplain colleague was not alone in rejecting a family’s request to pray for a miracle. Chaplain Jones ^26^ relates a similar experience from his early chaplaincy days. Jones tells the story of being asked by grief-stricken parents to pray for a miracle for their young daughter who was dying in a pediatric ICU:

A prayer was all that they had left, just a simple prayer for a miracle that their daughter would be healed and live. It was not too much to ask. But I could not pray for healing that night. See, I knew too much; or at least I thought I did at the time. ^26^

At the time, Jones saw the family’s hope for an unlikely miracle as an intellectual impasse. But in hindsight, Jones came to see his inability to petition God for the parents’ request as a form of emotional avoidance on his part; he confesses:

I could have prayed for a lot of things that night, but not for healing. It was better and safer to be theologically correct than to feel the depths of all the pain. It was easier to be theologically rigid than to admit to my helplessness. I was more interested in helping the parents through their denial and on toward acceptance. I knew my Kübler-Ross. I was learning clinical theory. My theology and interventional theory may have been right, but they were everso empty. ^26^

Jones summarizes his emotional shortcoming as a failure of compassion.

Not long ago, I found a journal entry from the time when I was contemplating the two extremes I had observed in my colleagues and trying to pinpoint where I fit on the spectrum constituted by their divergent approaches. Several other chaplains I spoke with shared one or the other of their perspectives or offered approaches that I also found unsatisfactory. One said that he felt pressure from the medical team to “deal with” these people, but he had no idea how to respond. Another said that she had developed language that allowed her to sound like she was praying for a miracle, even though that’s not really what she was praying for, so that she could appease people without offending them. That approach felt disingenuous to me.

Agonizing about where I stood on the spectrum of responses I had observed, especially in light of my relatively recent conversion to Eastern Orthodoxy, I found myself praying my way into a particular kind of prayer that synthesized aspects I admired about what I observed and what I struggled with in those two initial divergent approaches. One of my colleagues fully joined with the family in their hopes for a miracle. My other colleague fully faced the painful reality that the family was likely to encounter and offered to walk that difficult path with them. In both approaches, I saw a valiant response to a communication challenge, and I sought to somehow appropriate the good I saw in each approach.

In hindsight, what I think I struggled with in each response was its seeming need for an air of certainty that made me feel uncomfortable given the profound uncertainty characterized by the divergence between hope and expectation that seemed to define the situation. One response embraced the family’s hopes with what felt like an unrealistic air of positive certainty, and the other response rejected the family’s hopes with what seemed like an authoritarian air of negative certainty. I found myself struggling to communicatively embrace the family in their divergence of hope and expectation with a feebleness of uncertainty tinged with anticipatory grief that aligned with what I experienced as the reality of their situation.

Like my divinity school colleague, I endeavored to whole-heartedly enter into the family’s hopes for a miracle, no matter how unlikely, and partner with them in bringing that request to God in earnest. At first, this was not easy; I was a bit shaky, but it still felt right. I think part of my challenge was a battle between my head and my heart. We tell ourselves intellectually that it isn’t fair to the family to fan the flames of false hope, as if we are incapable of creative, nuanced, and transparent communication. But I wonder if that isn’t our own excuse for not wanting to enter that helpless space of painfully divergent hopes and expectations with them. True communicative “joining” is often painful; it requires “com”-“passion” where “com” means “with” and “passion” means “suffering.” Suffering *with* and being *with* suffering. As Jones confessed above, it “was better and safer” and “easier” to be “correct” and “rigid” and hold the family at arm’s length by refusing to pray for a miracle, rather than to “feel the depths of all the pain” and to “admit my helplessness,” which are prerequisites for joining them in crying out to God for the impossible.

Over time, I became surprisingly comfortable with suspending my calculations, embracing uncertainty, and trying to fully join the family in their hopes and offer those hopes up to God together with them. I found deep meaning in being with them right there in that space. Instead of wondering what kind of unrealistic expectations I may be creating by joining with them in their hopes, I harnessed the honesty and transparency that I admired in my chaplaincy training colleague who couldn’t bring himself to pray for a miracle, and I deliberately faced the uncertainty of the situation in the prayer itself rather than wrapping a neat little assumptive bow around everything and ending the prayer triumphantly, or even expectantly, as if the miracle was a forgone conclusion. For example, I named the reality that, far from assumptively claiming a positive outcome, what we are really doing in praying for a miracle is simply crying out to God as little children do to their parents, “lifting up” our hopes and cares and desires without calculation or pretense, and “laying them down at [his] feet”. ^27^ In praying for a little child to overcome a destructive brain tumor, I became a little child too and joined together with her parents as fellow children before the almighty God, lifting up their hopes with fear and trembling, with feebleness and fragility.

As a further effort to sincerely honor hope without creating false expectations, in addition to naming our child-like posture in prayer, I began to even more directly acknowledge the uncertainty of the situation in the prayer itself. In praying for a 58-year-old woman waiting in the transplant queue, troubled by the paradox that in hoping for a healthy heart to become available for her, she was effectively hoping for another healthy person to tragically die, I transparently named the uncertainty and ambivalence of the situation in the prayer, praying, “We have no idea what happens when we pray this prayer”. ^27^ Rather than ending with the focus on uncertainty or some false assertion of certainty about the corporeal, I ended these prayers with a focus on what we can know through spiritual experience—that God is always near to us, knowing our torment, and taking our torment into his heart; he was in the room, together with us, suffering with us and loving us. And even when God allows death, we can still affirm that death is not only an end to life on this earth; death is also a new beginning for eternal life in the presence of God.

Finding my way to this prayer transformed my experience of prayer requests for miraculous recovery from one of dread and terror to one of deep meaning. I felt that closeness that I was jealous of in the divinity student’s prayer, that feeling of being right there with the family. But I felt no concern about creating false expectations. Acknowledging the reality of uncertainty achieved the intellectual honesty that I respected in my other chaplain colleague. I was able to enter authentically into the family’s journey, to take this journey together. I sincerely hoped for their miracle. But if (as was most likely) the miracle was not to be, we could continue to express both our hopes and uncertainties and discover meaning together along the way.

Prayer that acknowledged the family’s heartfelt desire, the reality of uncertainty, and the certain presence of God’s loving embrace in this moment brought tremendous meaning to an otherwise desperate, impossible situation. I went from a sort of torture to deep joy in these encounters. And I noticed at the end of these prayers that there was often an emotional release. Even when the tears were accompanied by silence, those tears seemed to signify that permission was granted to hold on to hopes, and acknowledge fears, and feel pain all at once, rather than having to choose just one emotion in exclusion of the others. In bringing all these raw emotions to God, as the Psalmist did, there is a sort of naked authenticity that brings peace. Another thing I noticed was that, in situations where different members of the family disagreed over whether or not to remove the ventilator and whether or not to hope in God for a miracle, both parties would come to me individually and thank me for the prayer, saying that it was “exactly what was needed.” Here, too, there was a coming together in hope and uncertainty rather than taking sides.

This experience of feuding family members coming together over prayer makes me wonder if the response characterized by the kind of nuanced prayer I developed may be useful for physicians conducting “goals of care” conversations with families who are hoping for a miracle through divine intervention. What are the key takeaways from Spiritual Care experience in this context, and how might they apply to physician communication?

The first obvious lesson begins with a simple acknowledgement that responding to families hoping against all odds for a miracle via divine intervention is a complex communication challenge that requires a thoughtful, intentional response. While the challenge is real, it is also surmountable through caring, creative, and transparent communication. In sharing my story, I do not claim to offer an unprecedented spiritual intervention. I am certainly not the first Spiritual Care Provider to deepen my embrace of uncertainty and capacity for compassion by discovering that it is possible to sincerely hope and pray for a miracle while still facing the reality that it is unlikely. Chaplain Gardner’s account of “A day in the life of a Mayo chaplain” ^28^ is evidence enough that my general conclusions, while perhaps not the same as every other Spiritual Care Provider who has struggled in this context, are not unique to me. Gardner’s description of her practice includes both an earnest ^[^ ^1^ ^]^ prayer for miracles and a clearly articulated embrace of uncertainty, naming the difference between a hope/prayer and a promise.

The second lesson learned from Spiritual Care experience is that thinking you know too much to compassionately engage another’s hopes may be *an intellectual ruse concealing emotional avoidance.* While this communication challenge can first mask itself as an intellectual impasse, ^[^ ^2^ ^]^it has the potential to unfold into an emotional opportunity for a clinician to discover within him/herself a deeper level of compassionate communication in this critical healthcare context. ^[^ ^3^ ^]^

If the ruse of an intellectual impasse can get in the way of a breakthrough in compassionate communication for Spiritual Care Providers, how much more for physicians who are seldom heavily trained in the emotional dynamics of communication? But why are we turning the focus to physicians? Isn’t this properly the chaplains’ domain? Yes and no. While the spiritual realm may technically be the domain of Spiritual Care specialists, it is important to remember the clinical reality that it is the physicians who face the communication challenge created by the conflict between miracle hopes and medical limitations more often and more directly than anyone else. Because “goals of care” conversations unavoidably revolve around complex medical science, it is the physicians who spearhead these conversations. And because the purpose of this communication is to discern the proper “goals of care,” it is the physicians who encounter head-on the families whose medical decisions are influenced by their hopes for a miracle through divine intervention.

When a family’s hope for a miracle colors their response to a terminal prognosis and makes them resistant to physician recommendations for the withdrawal of aggressive measures, might prayer at that moment be helpful? Because I am suggesting a “point-of-care” prayer offered by the physician as part of the “goals of care” conversation, obvious challenges to this possibility come to mind. What if, as is often the case with so few chaplains in the hospital, no chaplain is present at that moment? Many physicians, regardless of their spiritual commitments, would not feel comfortable performing the prayer, even if they were given a text to read. This is a serious issue that would have to be worked out, but the advance of modern medicine is never halted by a challenge. Could communication technology be used to bridge this gap? We will return to that question below.

First, I want to emphasize that in raising the issue of physician-provided prayer, I am only marginally building upon a physician communication protocol already in existence. Thomas J. Smith, MD, Director of Palliative Medicine and Professor of Oncology at Johns Hopkins Hospital, in partnership with Hopkins Chaplain Cooper, Nurse Ferguson, and Dr. Bodurtha, proposed a communication protocol for clinicians to use in response to families hoping for a miracle when confronted with the limits of medical intervention to prolong life. ^29^ The “'AMEN' Protocol” is an acronym that stands for “affirm, meet, educate, no matter what.” Here is the protocol in brief:

• Affirm the patient's belief. Validate his or her position: “Ms. X, I am hopeful, too."
• Meet the patient or family member where they are: “I join you in hoping (or praying) for a miracle."
• Educate from your role as a medical provider: “And I want to speak to you about some medical issues.”
• No matter what; assure the patient and family you are committed to them: “No matter what happens, I will be with you every step of the way". ^29^

The first two steps of the protocol, “A” = “Affirm” and “M” = “Meet", come together to enact the vital process of “joining” that I referenced above in my personal story of learning to pray for miracles. First author, Cooper, shared with Cancer Therapy Advisor that a goal of the protocol is to communicatively empower the provider “to *join* [emphasis added] the patient or family member as a fellow human being with hopes and aspirations” which can build “a sense of trust and commitment to care". ^30^

Also key in the protocol is the use of the word “and” instead of the word “but” in the “E” = “ Educate” part of “AMEN,” as a transition from joining to educating. The AMEN Protocol authors stress that when physicians transition with the word “but” instead of the word “and,” they “unintentionally place themselves in direct competition with the God of the patient’s or family’s understanding". ^29^ So, if the physician says, “I join with you in hoping (or praying) for a miracle, *but* I want to speak to you about some medical issues,” they are diminishing their previous efforts at joining by introducing a spirit of competition. Thus, the recommendation to replace “but” with “and,” stating, “I join with you in hoping (or praying) for a miracle, *and* I want to speak to you about some medical issues.”

Although I know this is not the intention of the authors, I wonder if, for the patient/family involved, the AMEN Protocol in its current form might sometimes feel more like the physician is giving a nod to joining rather than actually joining wholeheartedly. I agree that transitioning with “and” rather than “but” definitely sounds less contentious. But if the goal of transitioning with “and” rather than “but” is to preserve a spirit of unity and prevent a spirit of disputation, is the swapping out of this one word really enough to prevent families from perceiving a transition from joining to debating?

I believe the AMEN Protocol is a giant step forward for providers communicating with families hoping for a miracle against all odds. Nevertheless, I wonder if simply stating, “I join you in hoping (or praying) for a miracle” before transitioning with “and I want to speak to you about some medical issues” accomplishes the stated goal of truly joining with suffering families and walking with them “every step of the way”. ^29^ What I fear is that, at least for some families, the “and” becomes a functional “but” when we give lip service to joining in hope or prayer without offering to communicatively perform that promise.

This is precisely what we witnessed in the *Extremis* documentary film quoted above. You will recall that, *true to the AMEN Protocol*, Dr. Zitter assured the African American family that the medical team, too, was hoping for a miracle. But the family remained unconvinced, perhaps because the doctor failed to back up her naked assertion with an actual offer to provide a prayer for the family’s hoped-for miracle. This anecdotal shortcoming in physician-provided spiritual care is consistent with population-based analyses performed by researchers. While most cancer patients, for example, desire spiritual care by physicians at the end of life, ^31^ they perceive the commonness of doctor-provided spiritual care to be “between 'never' and 'rarely'”. ^1^ This trend must change if we want to address the religious issues underlying racial and ethnic disparities in end-of-life healthcare.

Notably, Dr. Zitter’s practice with miracle-hoping African American families has evolved since the publication of her book and film. Confessing her previous discomfort staying in the room for a family prayer after breaking bad news, Zitter shared in a New York Times article that she now stays present for prayer at the encouragement of her hospital’s African American chaplain, Rev. Betty Clark. While staying in the room together with the family was uncomfortable for her at first, Dr. Zitter describes her presence during bedside prayer as a bridge of connection with African American families and recommends that her fellow physicians follow suit. ^32^ So moved is she by the profundity of her experience being present for third-party prayer with the family that she describes it as a reminder of what initially drew her to the medical vocation.

In parallel to the evolution of Dr. Zitter’s practice, I would like to suggest that the AMEN Protocol might be augmented through the embodiment of the “Affirm” and “Meet” steps if the physician offered to provide and stay respectfully present for a point-of-care, third-party prayer. If a chaplain who is comfortable praying sincerely for miracles is already present in the room or readily available, that would be ideal. Otherwise, out of necessity, one could resort to communication technology, as is commonly done for language translation in the case of Remote Video Interpreting.

Regardless of their own language of preference, it is common for physicians to work through a third-party translator to communicate with families in their own language. Similarly, regardless of their beliefs, the physician can honor the family’s suffering by offering to provide (via third party), and staying respectfully present for, a momentary prayer in hope that the family will benefit from their emotional support.

This analogy between remote, third-party, point-of-care, intercessory prayer and Remote Video Interpreting may be even stronger than it first appears. If, as we learned above, religious beliefs (and most saliently, the belief in miracles) statistically accounted for racial disparities between African Americans and white patients in end-of-life healthcare, ^13^ then showing what Harvard scholars, Balboni and Balboni, might call “hospitality” ^1^ to such beliefs is respecting both their culture and, in many cases, their ethnicity. Like providing language interpretation services via Remote Video Interpreting, it may be yet another form of *speaking their language.* It is no accident that the documentary video scenes referenced above depicted a white doctor struggling to speak a black family’s spiritual language. In the vein of endeavoring to speak the patient/family’s miracle language, Dr. DeLisser ^33^ encourages fellow physicians to engage and explore patient/family miracle hopes with curiosity, seeking to understand the “meaning and significance of a miracle” for each particular patient/family. I believe that in many cases, offering to provide a supportive prayer is an ideal first step in that direction, establishing the doctor as a trusted partner, so that patient/family values-exploration is experienced as support rather than interrogation or another tug of the rope in a tension-filled tug-of-war.

Krakauer, Crenner and Fox point out that too often it is patients and families who are held responsible for mistrusting their physicians, but the opposite should be true: “The onus should be on physicians and on the healthcare system to consistently demonstrate trustworthiness”. ^9^ Likewise, a review of palliative care issues identified by patients emphasizes the importance of religious support for African American patients and stresses that patient needs should dictate suitable interventions. ^34^ While palliative specialists deliver specialty palliative care, this intervention also falls under the umbrella of primary palliative care, which is also the responsibility of non-palliative physicians. In short, if the “AMEN” communication protocol for providers responding to miracle-hoping families included an actual “amen,” then I believe, as the old church saying goes, “all God’s children [would say] 'Amen.'”

## Discussion/conclusions

Point-of-care spiritual intervention is ethically advisable to help eliminate racial and ethnic disparities in end-of-life healthcare, but is it economically feasible to fund such a dramatic change in practice? Researchers contend that spiritual care by physicians at the end of life is infrequent for lack of training. ^31^ But research also suggests that there is a financial case to be made to pay for training. In one analysis, the medical team’s support of cancer patients’ spiritual needs was associated with an average of $2441 in healthcare cost savings ^[^ ^4^ ^]^ per patient in the last week of life. ^35^ This suggests that there may be a way to provision for both physician training and a remote setup for virtual, point-of-care spiritual intervention delivery given the return on investment for goal-adherent and value-based care. ^[^ ^5^ ^]^

This cost-savings finding turns common assumptions about supporting the miracle-hopes of African American families on their head. Rather than raising “false” hopes that could be counterproductive to their consideration of less aggressive medical decisions, supporting miracle hopes actually allows for more trusting *and more frank* discussions about the realities of commonly counterproductive and burdensome measures of intensive care at the end of life. This is seen in a study of racial disparities in physicians’ approach to end-of-life conversations. ^36^ While white doctors insisted: “These issues are colorblind” and reported the “need to stay 'indifferent'” to patients’ and families’ religious beliefs, African American doctors, in sharp contrast, insisted on the critical importance of identifying with African American patient’s/families’ religious beliefs and supportively engaging their hopes. African American doctors maintained that this approach allows them to establish the trust necessary to engage more fully with African American patient/family apprehensions and concerns surrounding end-of-life healthcare decisions. As a result, African American doctors were able to be *both* more supportive (saying things like, “I never try to squash anyone’s hopes”), *and also* “quite frank” and productively *carefrontational*, if you will, (saying things to the family like, “God doesn’t do miracles everyday”), all within a framework of respect for the family’s perspective that it is God who is ultimately in control (saying things like: “Now it’s up to God”). ^36^ This nuanced combination of 1) embracing hope, 2) naming difficult realities including uncertainty, and 3) embracing a faith that transcends unwanted outcomes, is reminiscent of the kind of prayer I found myself praying in these contexts and therefore the kind of spiritual intervention that I am advocating for doctors to offer via third-party specialists.

Some of the most profound spiritual experiences I have had in my life were mediated through encounters with African American women who self-identify as “prayer warriors” and stand out to me as spiritual giants. I envision a remote clearinghouse and/or work-at-home setup for many of these women, ^[^ ^6^ ^]^ trained for spiritual intervention in end-of-life contexts, virtually available to doctors on a point-of-care basis, to provide an earnest and nuanced prayer for miracle-hoping families. Seamlessly integrating this spiritual intervention into “goals of care” conversations with physicians is critical for three reasons. First exigency; this is needed on a point-of-care basis to prevent status quo harms to the physician-patient/family relationship and to physicians, patients, and families individually. ^10^^,^^11^^,^^15^^,^^16^^,^^20^^,^^21^ Second, we cannot leave this issue to church-based support alone. Many pastors and other church-based support personnel are not trained for this context; as a result: “Terminally ill patients who are well supported by religious communities access hospice less and aggressive medical interventions more near death”. ^37^ Third, the opposite is true for spiritual support *from the medical team.* Research demonstrates that: “Support of terminally ill patients’ spiritual needs by the medical team is associated with greater hospice utilization and, among religious copers, less aggressive care at [the end of life]”. ^38^

I believe that providing and staying respectfully present for a third-party prayer would allow white doctors who prize spiritual neutrality in these conversations to narrow the gap between their default preference for indifference and African American doctors’ contrasting default marshaling of their own spiritual resources. This would enable white doctors to serve the needs of patients and families while building trust in the physician-patient/family relationship and staying in their medical lane by leaving the actual prayer itself to a third-party spiritual specialist. We have seen that physician-patient/family conflicts over miracle hopes can result in a tension-filled tug-of-war, ^15^^,^^16^^,^^22^ and physician avoidance of miracle-hoping families (which only amounts to a passive-aggressive version of the same tug-of-war) can lead to complicated grief among families ^20^ and burnout among physicians. ^21^ In place of the direct tug-of-war of disputing the family’s hopes or the indirect tug-of-war of avoidance, I am advocating for physicians to drop their end of the tug-of-war “rope” of conflict, walk across the divide to meet families where they stand, on the other side of the rope, and traverse across the bridge together, hand-in-hand. As seen in the narrative arc of Dr. Zitter’s documented experience, ^22^^,^^32^ this will reduce the unwanted outcomes of undue stress in these contexts and result in the deeper therapeutic alliance desired by all involved.

In addition to the statistically appropriate focus on miracle beliefs in African American families (one analysis found that 96% of African American patients subscribed to the belief that “God can perform a miracle and cure me” ^13^), it must also be admitted that these beliefs can manifest in any and every race/ethnicity. The spiritual intervention protocol herein advocated, then, much like the targeted advance-care-planning interventions advocated by Garrido, Harrington, and Prigerson, ^39^ may *both*, 1) “reach a broad patient population” *and also*, 2) “reduce end-of-life care disparities."

A closing anecdote: Several years ago, after delivering a presentation on the paradox of “false” hope for Grand Rounds at a leading academic medical center, I was approached afterwards by a palliative care social worker who relayed a salient story. The previous week, she recalled debriefing with a physician who vented his frustration coming out of a “goals of care” conversation, stating with notable dismay, “the family is just fixated on their miracle.” When the medical team later returned for their next conversation with the family, the social worker brought the chaplain along and began the conversation by asking the chaplain to offer a prayer. Immediately after the prayer, the family raised their gaze to meet the physician and, to his great surprise, expressed their readiness to withdraw the ventilator. “All they wanted,” exclaimed the social worker, “was a prayer.”

What may first seem like an irreconcilable conflict between science and religion does not need to be reduced to an exclusively intellectual impasse. I believe that caring communication can transform seemingly irreconcilable differences into surprisingly meaningful alliances. This does not mean that prayer will always produce a magical agreement between the physician and the family on the proper goals of care. Nevertheless, is there any harm in replacing emotional avoidance with compassionate communication and seeing where it might lead?

## Data availability

### Underlying data

No data are associated with this article.

## Footnotes

^1^
I can only assume.

^2^
Recall Jones’ words above: “See I knew too much; or at least I thought I did at the time.”

^3^
In hindsight, Jones specifically states, “All I needed was *compassion.*” While traveling a different route (I never denied the prayer as Jones did) I came to a similar conclusion about compassion being the key to moving from my head to my heart.

^4^
As stated in the introduction, at the end of life, more intensive, death-prolonging care tends not to be better as aggressive measures at the end of life tend to be as physically and emotionally costly to patients and families as they are financially costly [4, 5 as cited in 1]. As a result, the financial cost savings in the last week of life that is associated with spiritual support from the medical team is not a motivating conflict of interest but rather a coincidental alignment of interests as an indicator of a less burdensome and more peaceful death.

^5^
Such a service is not to replace hospital chaplains but to increase exigent chaplaincy referrals for follow up.

^6^
Together with other race/gender/religion profiles to meet diversity standards and serve all interested patients and familes.
