# Supplementary material for: Honoring Black Hopes: How to respond when the family is hoping for a miracle
Source: F1000Res. 2022 Mar 2;11:268. [Version 1] doi: 10.12688/f1000research.109811.1 (PMC9345262; doi:10.12688/f1000research.109811.1)
Supplement: Peer review history

# Peer review: Reviewer response for version 1

**DOI:** 10.5256/f1000research.121359.r141542

**Published:** 2022-08-01

**Authors:** Maria Leonor Antunes

**Affiliations:** 1 Hospital do Espírito Santo, Évora, Portugal

The article addresses an important issue within spiritual care: how to address African American families’ hope for a miracle in end-of-life healthcare. The methodology used is autoethnographic, a qualitative method of research that requires deep reflection on one’s unique experience, using the researcher’s insights to interpret cultural and social phenomena.

 Even though this methodology has some limitations, such as the risk of researcher bias, the complex phenomenon involved in spiritual care requires deep inner reflection, that often cannot be adequately conveyed by quantitative methods of research. Qualitative approaches frequently offer useful and tangible contributions, that can inspire the reader to reflect on his/her own approach to patients.

 In this paper, the author guides the reader through his own personal journey in caring for African American families who base end-of-life medical decisions on their hopes for a miracle. I believe it provides a significant contribution to previous literature on this topic, offering new resources to physicians when pre-existing protocols for communication fail. The author’s reflection and deep theoretical embedding provide hope for physicians who wish to provide compassionate care, “suffering *with*” the ones who suffer, but struggle with the fear of fueling unrealistic hopes.

 The article presents a different approach, one that allows healthcare providers to wholeheartedly join the family’s prayer for a miracle, without creating false expectations. It is competently executed, with a clear exposition of the author’s experiences, which he combines with the relevant literature. I would deem this paper a must-read for practitioners of the field.

 I hope this article can be used as a tool for healthcare professionals’ spiritual care training. I’m also looking forward to evidence-based future articles of how patients and physicians experience this approach.

Is the work clearly and accurately presented and does it cite the current literature?

Yes

If applicable, is the statistical analysis and its interpretation appropriate?

Not applicable

Are all the source data underlying the results available to ensure full reproducibility?

No source data required

Is the study design appropriate and is the work technically sound?

Yes

Are the conclusions drawn adequately supported by the results?

Yes

Are sufficient details of methods and analysis provided to allow replication by others?

Yes

Reviewer Expertise:

The physician's role in spiritual care provision. Development of educational tools for healthcare professionals' training in spiritual care.

I confirm that I have read this submission and believe that I have an appropriate level of expertise to confirm that it is of an acceptable scientific standard.

# Peer review: Reviewer response for version 1

**DOI:** 10.5256/f1000research.121359.r135047

**Published:** 2022-05-09

**Authors:** Ronit Elk

**Affiliations:** 1 Division of Geriatrics, Gerontology, and Palliative Care, Department of Medicine, School of Medicine, University of Alabama, Birmingham, AL, USA

A very relevant paper that addresses an issue that is very prevalent: lack of physician understanding of, and respect for, African American families' hope for a miracle at end of life. The method is autoethnographic, a form of self-reflection and writing that explores the researcher's personal experience and connects this autobiographical story to wider issues. In this paper it is the author’s spiritual care experience on grappling with the issue of honouring Black hopes when the family’s loved one is seriously ill. This paper provides an excellent background of publications relevant to this issue. The conclusions and recommendations are based on the combination of the literature and the autoethnographic reporting. I hope this paper is (1) accompanied by an editorial in the journal and (2) is shared with hospice and palliative care providers.

Is the work clearly and accurately presented and does it cite the current literature?

Yes

If applicable, is the statistical analysis and its interpretation appropriate?

Not applicable

Are all the source data underlying the results available to ensure full reproducibility?

No source data required

Is the study design appropriate and is the work technically sound?

Yes

Are the conclusions drawn adequately supported by the results?

Yes

Are sufficient details of methods and analysis provided to allow replication by others?

Yes

Reviewer Expertise:

End of life care in African Americans. Community based participatory research to build palliative care programs based on the cultural values of underrepresented communities.

I confirm that I have read this submission and believe that I have an appropriate level of expertise to confirm that it is of an acceptable scientific standard.
